# A Variant in the *BACH2* Gene Is Associated With Susceptibility to Autoimmune Addison's Disease in Humans

**DOI:** 10.1210/jc.2016-2368

**Published:** 2016-09-28

**Authors:** Agnieszka Pazderska, Bergithe E. Oftedal, Catherine M. Napier, Holly F. Ainsworth, Eystein S. Husebye, Heather J. Cordell, Simon H. S. Pearce, Anna L. Mitchell

**Affiliations:** Institute of Genetic Medicine (A.P., C.M.N., H.F.A., H.J.C., S.H.S.P., A.L.M.), Newcastle University, Newcastle upon Tyne NE1 3BZ, United Kingdom; Department of Clinical Science (B.E.O., E.S.H.), University of Bergen, 5021 Bergen, Norway; and Department of Medicine (E.S.H.), Haukeland University Hospital, 5021 Bergen, Norway

## Abstract

**Context:**

Autoimmune Addison's disease (AAD) is a rare but highly heritable condition. The BACH2 protein plays a crucial role in T lymphocyte maturation, and allelic variation in its gene has been associated with a number of autoimmune conditions.

**Objective:**

We aimed to determine whether alleles of the *rs3757247* single nucleotide polymorphism (SNP) in the *BACH2* gene are associated with AAD.

**Design, Setting, and Patients:**

This case-control association study was performed in two phases using Taqman chemistry. In the first phase, the *rs3757247* SNP was genotyped in 358 UK AAD subjects and 166 local control subjects. Genotype data were also available from 5154 healthy UK controls from the Wellcome Trust (WTCCC2) for comparison. In the second phase, the SNP was genotyped in a validation cohort comprising 317 Norwegian AAD subjects and 365 controls.

**Results:**

The frequency of the minor T allele was significantly higher in subjects with AAD from the United Kingdom compared to both the local and WTCCC2 control cohorts (58% vs 45 and 48%, respectively) (local controls, *P* = 1.1 × 10^−4^; odds ratio [OR], 1.68; 95% confidence interval [CI], 1.29–2.18; WTCCC2 controls, *P* = 1.4 × 10^−6^; OR, 1.44; 95% CI, 1.23–1.69). This finding was replicated in the Norwegian validation cohort (*P* = .0015; OR, 1.41; 95% CI, 1.14–1.75). Subgroup analysis showed that this association is present in subjects with both isolated AAD (OR, 1.53; 95% CI, 1.22–1.92) and autoimmune polyglandular syndrome type 2 (OR, 1.37; 95% CI, 1.12–1.69) in the UK cohort, and with autoimmune polyglandular syndrome type 2 in the Norwegian cohort (OR, 1.58; 95% CI, 1.22–2.06).

**Conclusion:**

We have demonstrated, for the first time, that allelic variability at the *BACH2* locus is associated with susceptibility to AAD. Given its association with multiple autoimmune conditions, *BACH2* can be considered a “universal” autoimmune susceptibility locus.

## Abstract (precis)

We performed a two-stage case control association study in UK and Norwegian cohorts and found that allelic variability in BACH2 locus confers susceptibility to autoimmune Addison's disease.

The BACH2 transcription factor (BTB and CNC Homology 1, Basic Leucine Zipper Transcription factor 2) is expressed predominantly in B lymphocytes and plays a vital role in regulating CD^4+^ T-cell differentiation. Its functions include repressing effector CD^4+^ T-cell lineages (Th1, Th2, and Th17), and it is crucial for the formation of regulatory T cells (1). It is therefore a key modulator of inflammation, keeping immune system activation in check and controlling the balance between immunity and tolerance. The *BACH2* gene is located on chromosome 6q15. In murine models, disruption of the *BACH2* gene results in mice that are phenotypically normal at birth, but which develop fatal autoimmune disease in the first months of life (1). In humans, a number of common variants in linkage disequilibrium (LD) at the *BACH2* locus have been consistently associated with autoimmune conditions, including type 1 diabetes (2), celiac disease (3, 4), autoimmune thyroid disease (5, 6), Crohn's disease (7), multiple sclerosis (8), vitiligo (9), and rheumatoid arthritis (10). Variants in the *BACH2* gene have yet to be studied in autoimmune Addison's disease (AAD), which is a rare but highly heritable, organ-specific autoimmune condition with a prevalence in the European Caucasian population of 110–220 cases per million (11, 12).

We aimed to determine whether the intronic single nucleotide polymorphism (SNP) *rs3757247* is associated with AAD in two independent cohorts from the United Kingdom and Norway. This SNP was selected for investigation because it has previously been associated with type 1 diabetes and generalized vitiligo, with odds ratios of 1.13 and 1.21 for the T allele conferring disease susceptibility, respectively (9, 13), and it is in tight LD (r^2^ > 0.93) with SNPs *rs11755527* and *rs619192*, which have been associated with type 1 diabetes (2), and *rs72928038*, which has been associated with rheumatoid arthritis (10) (Figure 1). In addition, it is in moderate LD (r^2^ = 0.4) with *rs10806425*, which has been associated with celiac disease (3).

**Figure 1.**
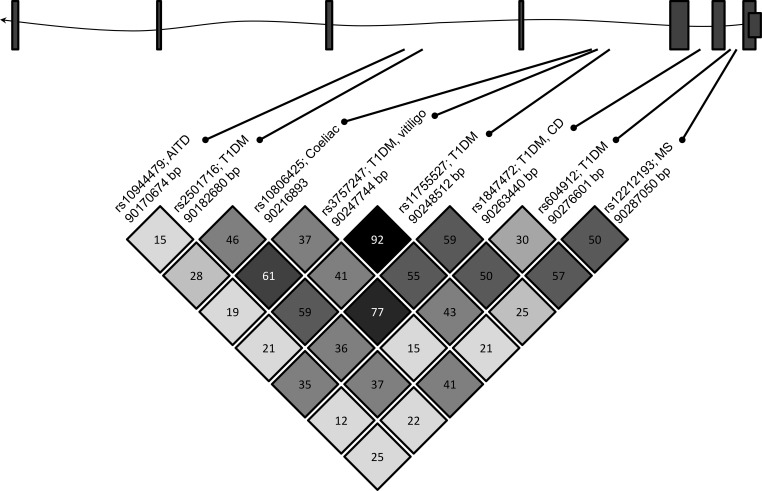
Schematic representation of the BACH2 locus and pairwise LD r^2^ measures between selected BACH2 SNPs previously associated with autoimmune conditions. At the top of the figure, a diagrammatic representation of the *BACH2* gene is shown. Exons are denoted by boxes, and intronic sequence by the line. In the lower half of the figure, SNPs previously associated with autoimmune conditions are identified by their unique rs numbers. The autoimmune diseases associated with each SNP are shown (AITD, autoimmune thyroid disease; T1DM, type 1 diabetes; MS, multiple sclerosis; CD, Crohn's disease), as is the base pair position of each SNP. Arrows mark the approximate location of each SNP relative to the introns and exons.

## Subjects and Methods

This study was carried out with approval of the Leeds East ethics committee (Ref. 05/Q1206/144) and the Regional Committee for Medical and Health Research Ethics (Ref. 2013/1504/REK vest).

The UK AAD cohort comprised 365 Caucasian individuals, of whom 285 were female. A diagnosis of AAD was confirmed by the subjects having either a low basal cortisol level with a high ACTH level or a subnormal response to the short synacthen test (250 mg parenteral synthetic ACTH_1–24_). Patients with primary adrenal failure due to adrenal gland infiltration or infection, with secondary adrenal failure, or with autoimmune polyglandular syndrome type 1 (on the clinical grounds of mucocutaneous candidiasis, hypoparathyroidism, and/or ectodermal dystrophy) were excluded. The median age at diagnosis was 39 years (range, 10–83 years). In 197 (54%) patients, an additional autoimmune condition (termed autoimmune polyglandular syndrome type 2 [APS2] was present, whereas 168 (46%) patients had AAD alone (termed isolated AAD [iAAD]). For comparison, a local matched control cohort comprising 183 individuals was available. In addition, genotype data from 5159 healthy individuals was available through the Wellcome Trust Case Control Consortium 2 (WTCCC2), as previously described (14).

We used 330 Norwegian AAD subjects, of whom 215 were female, and 384 matched controls as an independent replication cohort. The diagnosis of AAD was made using the criteria described above.

Genomic DNA was extracted from venous blood for each subject in the AAD and local control cohorts. The *rs3757247* SNP was then genotyped using Taqman chemistry (Life Technologies, Thermo Fisher Scientific) according to the manufacturer's instructions. Ten percent of the samples were genotyped in duplicate to ensure accuracy of results. Genotype results were available for 358 of 365 UK AAD subjects (genotyping call rate, 98%) and for 166 of 183 local UK controls (genotyping call rate, 91%). Genotyping call rates in the Norwegian AAD subjects and the Norwegian control cohorts were 96% (317 of 330) and 95% (365 of 384), respectively. Genotypes were checked for Hardy-Weinberg equilibrium in both control cohorts (threshold *P* > .05) before analysis.

Statistical analysis was performed using PLINK, a freely available association analysis engine (15). A heterogeneity analysis was performed to compare genotype and allele frequencies between the UK control cohort and the WTCCC2 controls to ensure that they were comparable. Association analysis was then performed between case and control cohorts, followed by a subgroup analysis comparing genotype and allele frequencies between the iAAD and APS2 subgroups from the United Kingdom compared to the WTCCC2 controls, and between the iAAD and APS2 subgroups from Norway and the Norwegian controls.

## Results

The genotype frequencies in the control cohorts were in Hardy Weinberg equilibrium (*P* > .05). There was no significant difference between genotype and allele frequencies between the UK local control cohort and the WTCCC2 controls (*P* = .24 and .21, respectively), and therefore these control cohorts were deemed comparable (Table 1).

**Table 1.** Comparison of Genotype and Allele Frequencies Between the UK Local and WTCCC2 Cohorts

Cohort	Call Rate (%)	Genotype Data				Allele Data		
		TT (%)	TC (%)	CC (%)	*P* Value	T (%)	C (%)	*P* Value
Local controls	166/183 (91)	36 (22)	77 (46)	53 (32)	.24	149 (45)	183 (55)	.21
UK WTCCC2	5154/5159 (100%)	1178 (23)	2630 (51)	1346 (26)		4986 (48)	5322 (52)	

Comparing the UK AAD cohort to the local controls, the frequency of the TT genotype was higher, whereas the frequency of the CC genotype was lower in AAD subjects (*P* = .0009) (Table 2). The minor T allele accounted for 413 alleles (58%) in the AAD cohort compared to 149 alleles (45%) in the control cohort (*P* = .00011; odds ratio [OR], 1.68; 95% confidence interval [CI], 1.29–2.18) (Table 2).

**Table 2.** Genotype and Allele Frequencies for the UK and Norwegian AAD and Control Cohorts

Cohort	Call Rate (%)	Genotype Data				Allele Data			
		TT (%)	TC (%)	CC (%)	*P* Value	T (%)	C (%)	*P* Value	OR [95% CI]
UK AAD	358/365 (98)	124 (35)	165 (46)	69 (19)		413 (58)	303 (42)		
UK local controls	166/183 (91)	36 (22)	77 (46)	53 (32)	9 × 10^−4a^	149 (45)	183 (55)	1.1 × 10^−4a^	1.68^a^ [1.29–2.18]
UK WTCCC2	5154/5159 (100)	1178 (23)	2630 (51)	1346 (26)	9.98 × 10^−7b^	4986 (48)	5322 (52)	1.40 × 10^−6b^	1.44^b^ [1.23 to 1.69]
Norwegian AAD	317/330 (96)	83 (26)	161 (51)	73 (23)	.003^c^	327 (52)	307 (48)	.0015^c^	1.41^c^ [1.14–1.75]
Norwegian controls	365/384 (95)	76 (21)	162 (44)	127 (35)		314 (43)	416 (57)		

a UK AAD vs UK local controls.

b UK AAD vs UK WTCCC2.

c Norwegian AAD vs Norwegian controls.

Comparing the AAD cohort to the larger WTCCC2 controls, a similar association was seen. The frequency of the TT genotype was higher, whereas the frequency of the CC genotype was lower in AAD cases (*P* = 9.98 × 10^−7^) (Table 2). The minor T allele accounted for 413 alleles (58%) in the AAD cohort compared to 4986 (48%) in the control cohort (*P* = 1.4 × 10^−6^; OR, 1.44; 95% CI, 1.23–1.69) (Table 2).

The above findings were replicated in an independent cohort of AAD and control subjects from Norway. In this group, the TT genotype was found in 83 AAD cases (26%) compared to 76 controls (21%), whereas the CC genotype was present in 73 AAD subjects (23%) and in 127 controls (35%). A total of 161 AAD subjects (51%) were heterozygous compared to 162 controls (44%) (*P* = .003). The minor T allele accounted for 327 alleles (52%) in the AAD cohort and 314 alleles (43%) in the control population (*P* = .0015; OR, 1.41; 95% CI, 1.14–1.75) (Table 2).

We conducted a subgroup analysis in the two cohorts by dividing the cases into those with APS2 and iAAD (Table 3). In the UK APS2 cohort, the T allele was present in 221 cases (57%) compared to 4986 controls (48%) (*P* = .0013; OR, 1.37; 95% CI, 1.12–1.69). Comparing the UK iAAD subgroup to the WTCCC2 controls (Table 3), a similar trend with stronger association was observed, with the T allele being present in 192 (59%) of the cases compared to 4986 (48%) controls (*P* = .00018; OR, 1.53; 95% CI, 1.22–1.92). In the Norwegian cohort, in the APS2 subgroup, the T allele was present in 182 (54%) cases compared to 314 (43%) controls (*P* = .00049; OR, 1.58; 95% CI, 1.22–2.06). In the iAAD subgroup, there was nominal association with genotypes (*P* = .04), but no association was detected with alleles (*P* = .12) (Table 3).

**Table 3.** Genotype and Allele Frequencies for the UK and Norwegian Subgroup Analysis

Cohort	Genotype Data				Allele Data			
	TT (%)	TC (%)	CC (%)	*P* Value	T (%)	C (%)	*P* Value	OR [95% CI]
UK iAAD	57 (35)	78 (48)	28 (17)	.0005^a^	192 (59)	134 (41)	1.8 × 10^−4a^	1.53^a^ [1.22–1.92]
UK APS2	67 (34)	87 (45)	41 (21)	.0009^b^	221 (57)	169 (43)	1.3 × 10^−3b^	1.37^b^ [1.12–1.69]
UK WTCCC2	1178 (23)	2630 (51)	1346 (26)		4986 (48)	5322 (52)		
Norwegian iAAD	31 (21)	83 (55)	36 (24)	.0369^c^	145 (48)	155 (52)	.12^c^	1.24^c^ [0.95–1.62]
Norwegian APS2	52 (31)	78 (47)	37 (22)	.0038^d^	182 (54)	152 (46)	4.9 × 10^−4d^	1.58^d^ [1.22–2.06]
Norwegian controls	76 (21)	162 (44)	127 (35)		314 (43)	416 (57)		

UK iAAD and APS2 are compared to the WTCCC2 cohort, and Norwegian iAAD and APS2 are compared to local controls.

a UK iAAD vs UK WTCCC2.

b UK APS2 vs UK WTCCC2.

c Norwegian iAAD vs controls.

d Norwegian APS2 vs controls.

## Discussion

This is the first report of a *BACH2* variant being associated with susceptibility to AAD. The association observed in the UK cohort was replicated in an independent cohort from Norway. *BACH2* variants have previously been associated with the more common autoimmune endocrinopathies, type 1 diabetes and autoimmune thyroid disease. In this study, the T allele at *rs3757247* appears to be conferring a greater risk in AAD compared to type 1 diabetes and vitiligo (OR, 1.44 and 1.41 in UK and Norwegian AAD cohorts, respectively; compared to 1.13 in type 1 diabetes and 1.21 in generalized vitiligo). Functional studies are now required to investigate the precise mechanisms by which variants in the *BACH2* gene confer susceptibility to autoimmune disease.

We have found that the intronic SNP, *rs3757247*, is associated with both iAAD and APS2 in the UK cohort, suggesting that this variant is independently associated with AAD in the UK population and that the association observed is not simply due to the fact that this cohort is enriched for those with additional autoimmune comorbidities. In the Norwegian cohort, however, there was association with the cohort as a whole, and in the subgroup analysis, with APS2. This suggests some genetic heterogeneity between these two European cohorts as has previously been reported (16).

To increase the power of this study, the UK AAD cohort was compared to the genotype data available from the WTCCC2 controls. The samples included in the WTCCC2 were genotyped using a SNP array. To control for the use of different genotyping platforms and to ensure that the WTCCC2 results were comparable to those derived from local controls, we compared genotypes and allele frequencies derived from a local UK control cohort to those from the WTCCC2 controls. This analysis demonstrated no significant differences between those two control groups.

How *BACH2* variants influence autoimmune disease susceptibility is not yet understood. However, possible mechanisms can be proposed on the basis that the BACH2 protein has a number of crucial functions in modulating inflammation and immunity. The BACH2 protein is known to play an important role in regulating lymphocyte differentiation. It has been shown to repress genes that are important for both Th1 and Th2 lineage differentiation, including *GATA3*. GATA3 is the master Th2 lineage transcription regulator, and polymorphisms in the *GATA3* gene have previously been associated with AAD (16–18). Indirect evidence, although limited, supports the role of IFN-γ, the Th1-derived cytokine, in adrenocortical destruction in AAD. The expression of major histocompatibility complex class II molecules on adrenocortical cells has been shown to be highly up-regulated during the active phase of AAD; this effect is likely mediated by IFN-γ (19, 20). In addition, T cells derived from AAD patients demonstrate higher IFN-γ production after stimulation with 21-hydroxylase compared with healthy controls (21). Our finding that genetic variability in the *BACH2* gene is associated with susceptibility for AAD further implicates this immune pathway in the etiopathogenesis of this condition.

This study has identified that a *BACH2* polymorphism is associated with AAD in two independent European cohorts. The *rs3757247* SNP is a common intronic variant, has no known functional consequences, and therefore is not causative of autoimmune disease. It is likely that this SNP is in LD with a causative variant located elsewhere in the locus.

The role of rare variants, with minor allele frequencies of <0.01, in complex traits has been increasingly recognized (22). A number of rare variants have been reported in the *BACH2* gene, and some of these are predicted to be potentially deleterious. A review of SNP data available from the 1000 Genomes project (23), visualized on the freely available Ensembl genome browser (release 85) (24), has shown that five rare missense variants at the *BACH2* locus are predicted to be deleterious by both SIFT and Polyphen analysis. These are located in a 20-kB region of the *BACH2* gene, with three located in exon 7 and one located in each of exons 8 and 9. These rare missense variants are located over 250 kB from the intronic SNP analyzed in this study and are not predicted to be in tight LD with *rs3757247*. A single-point analysis of the rare variants in the *BACH2* gene in our cohort would be underpowered because of the low copy number of the minority allele; however, they warrant further investigation. The exact mechanism by which *BACH2* variants influence autoimmune disease risk requires further research, and a systematic analysis of variation in the region is now required in large cohorts of patients. Our results add to the growing literature that demonstrates that the BACH2 protein is a crucial regulator of both immune function and dysfunction. Autoimmune diseases are known to share a common genetic architecture, with some loci, such as *CTLA4*, *PTPN22*, and the *HLA*, conferring susceptibility to multiple autoimmune diseases. The finding of association of a *BACH2* variant with a further autoimmune condition, AAD, supports the hypothesis that variation in *BACH2* may be a permissive immune system factor that is implicated in many or most organ-specific autoimmune conditions.
